# Association between Musculoskeletal Pain and Bone Turnover Markers in Long-Term Pb-Exposed Workers

**DOI:** 10.34172/jrhs.2021.55

**Published:** 2021-07-06

**Authors:** Ravibabu Kalahasthi, Bhavani Shankara Bagepally, Tapu Barman

**Affiliations:** ^1^Department of Biochemistry, Regional Occupational Health Centre (Southern), Indian Council of Medical Research, ICMR Complex, Karnataka, India; ^2^Department of NCD, National Institute of Epidemiology, Chennai, India; ^3^Department of Bacteriology, National Institute of Cholera and Enteric Diseases, Beleghata, Kolkata, West Bengal, India

**Keywords:** Musculoskeletal pain, Bone remodeling, Bone formation, Bone resorption, Lead, Blood, Occupational exposure

## Abstract

**Background:** On chronic exposure, Lead (Pb) deposits in the skeletal system, replaces calcium ions, and alters the normal physiological processes, which in turn, lead to stunting, delayed fracture healing, and high resorption of collagen molecules. The present study aimed to assess the association of musculoskeletal pain and discomfort with bone turnover markers (BTMs) among long-term Pb-exposed workers.

**Study design:** A cross-sectional study.

**Methods:** The study recruited 176 male Pb-exposed workers and 80 control subjects who were matched for age, gender, and socio-economic status. Blood lead levels (BLLs), bone growth markers, such as serum osteocalcin (OC), alkaline phosphatase (ALP), bone alkaline phosphatase (BAP), and bone resorption markers: serum pyridinoline (Pry), deoxypyridinoline (DPry), tartrate-resistant acid phosphatase-5b(TRACP-5b), and hydroxyproline in urine (HyP-U) of participants were investigated. Pain and discomfort in the musculoskeletal system were assessed using Nordic Musculoskeletal Questionnaire.

**Results:** Pb-exposure was significantly associated with musculoskeletal discomfort of the lower back (P<0.001), upper back (P<0.001), and ankle/foot (P=0.011). Among bone formation markers, serum OC was significantly lower in musculoskeletal discomfort of elbows (P=0.033) and ankle/foot (P=0.042). Among bone resorption markers, serum DPry was significantly lower in musculoskeletal discomfort of the neck (P=0.049) and shoulders (P=0.023). HyP-U was significantly higher in musculoskeletal discomfort of shoulders (P=0.035) and lower back (P=0.036).

**Conclusion:** As evidenced by the obtained results, Pb-exposure was associated with musculoskeletal discomfort of the lower back, upper back, and ankle/foot. Lower bone formation (serum OC) marker was noted with musculoskeletal discomfort of elbows and ankle/foot. Furthermore, bone resorption markers were associated with musculoskeletal discomfort of the neck, shoulders, and lower back. The findings of the present study suggested that long-term Pb-exposure and BTMs were associated with musculoskeletal discomfort.

## Introduction

Humans are occupationally exposed to lead (Pb) in battery manufacturing plants, Pb-smelting, Pb-recycling, as well as lead-based paints and pigments^1^. The main route of entry in occupational Pb exposure is through inhalation, and it is accumulated in soft tissues, such as erythrocytes, liver, and kidney, as well as hard tissues, namely the skeletal system and teeth. The skeletal system is required for vital movement, body support, and organ protection^2^. In Pb exposure, the highest percentage of Pb is accumulated in the bone matrix which acts as the chief target organ. It has the ability to displace divalent metal ions (Zn2+, Ca2+, Mg2+&Fe2+) in proteins and a high binding affinity of thiol groups in active sites of enzymes. These Pb toxicity mechanisms contribute greatly to reduced skeletal growth and delayed fracture healing ^3,4^. Female workers in Pb-smelting and Pb-battery plants may have higher bone resorption with an increased risk of osteoporosis ^5,6^. Among Pb-battery workers, a review of the literature reported altered bone turnover markers (BTMs)^7^, diminished bone mineral density (BMD)^8^, and a threat to osteoporosis ^9^. Middle-aged workers reported a positive association between Pb-exposure and calcified cartilage turnover markers ^10^.

The evaluation of BTMs recommends the bone remodeling status, comprising bone formation and bone resorption activities ^11^. Bone formation markers are used to assess the osteoblast cell activity, including alkaline phosphatase (ALP), bone alkaline phosphatase (BAP), osteocalcin (OC), and procollagen type1 N propeptide (PINP). Bone resorption markers are employed to determine osteoclast activity. These markers are the degradation products of collagen molecules, including tartrate-resistant acid phosphatase-5b (TRACP-5b), DPry, Pry, UHyP, C-terminal cross-linking telopeptide of type I collagen (CTX-I), and N-terminal cross-linking telopeptide of type I collagen (NTX-I). The evaluation of BTMs has been used to indicate bone loss, risk of fracture, and treatment efficacy of osteoporosis medications ^12^. Recent reviews of the literature indicated that BTMs were associated with femoral neck size and strength ^13^, BMD ^14^, risk of hip fracture ^15^, skeletal histiocytosis ^16^, heel stiffness index ^17^, neck circumference ^18^, rheumatoid arthritis ^19^, and knee osteoarthritis ^20^.

The MSDs are injuries or pain in the musculoskeletal system ^21^, and work-related MSDs result from exposure to work-related risk factors ^22^. The literature review suggested that the musculoskeletal system is vulnerable to Pb-toxicity, even at low levels of Pb-exposure, affecting the motor skills, bone growth and development, dentition, bone density, fracture healing, and joint functions ^23^. Bone accounts for 94% of the body burden of Pb in adults ^24^. Animal studies suggested that Pb-intoxication leads to delayed fracture healing and fibrous nonunion due to the progression of endochondral ossification ^25^. Occupational activities, such as repetitive overload, can cause bone loss and micro-cracks with an increase in receptor activator of nuclear factor-kappa-Β ligand (RAKL) and sclerostin ^26^. The serum concentration of carboxyl-terminal propeptide of type I collagen (PICP) and CTx is associated with a heavy physical workload among construction workers ^27^. Lower limb MSDs were associated with urinary c-telopeptide of collagen II ^28^. A recent study also reported that Pb-exposure and inflammatory markers were significantly associated with lower limb MSDs ^29^.

 Nonetheless, there is a dearth of studies examining the effect of chronic occupational Pb exposure on pain and discomfort in the musculoskeletal system, as well as its association with biochemical parameters, such as BTMs. Therefore, the present study aimed to explore the association of (a) BLLs (Pb-exposure) with pain and discomfort in the musculoskeletal system and (b) pain and discomfort in the musculoskeletal system and BTMs (formation and resorption) among the workers of Pb-battery plants. Furthermore, this study conducted a stratified analysis to explore the association of Pb-exposure with pain and discomfort of the upper body (neck, shoulder, and upper back), upper extremity (elbows and wrist/hands), lower extremity (thighs, ankle/foot, and knees), and BTMs.

## Methods

 This cross-sectional analytical study was conducted on 256 participants during 2014-2015. The subjects were assigned to two groups of study and control. The study group consisted of 176 male workers who were occupationally exposed to Pb for more than two years in a Pb-battery manufacturing plant situated in Tamil Nadu, India. The control group encompassed 80 office workers with no occupational exposure to Pb. The subjects in the study and control groups were matched for age and socioeconomic status. The Ethics Committee of the Regional Occupational Health Centre (Southern) approved the present study. The subjects were informed of the objectives of the study and signed written informed consent before their participation. Demographic information and a chronological list of employment was obtained, and individual habits of subjects were collected using a structured questionnaire.

###  Pain and discomfort in the musculoskeletal system

The Nordic musculoskeletal questionnaire (NMQ) was employed to assess pain and discomfort in the musculoskeletal system of study and control subjects ^30^. The NMQ is a validated questionnaire used to assess the frequencies of pain and discomfort in different parts of the musculoskeletal system^31^.

###  Collection of samples

From each subject, 2 ml heparinized and 3 ml whole blood samples were collected in tubes. Moreover, 50 ml of urine sample was collected from each subject. In this regard, 2 ml of heparinized whole blood sample was used for the estimation of BLLs, and 3 ml of whole blood collected in plain tubes was centrifuged at 3000 RPM for 10 min at 4^o^C for the separation of serum and blood cells. The serum sample was used for the estimation of BTMs. The collected urine sample was utilized for hydroxyproline (HyP) and creatinine determinations.

###  BLLs

The BLLs were measured as described by Barman et al. ^32^. In this protocol, 2 ml of heparinized whole blood sample was digested using an ETHOS-D microwave digestion system with 2 ml of nitric acid (HNO_3_) and 0.2 ml of hydrogen peroxide (H_2_O_2_). The digested samples were made up to 5 ml using distilled water. The BLLs concentration was measured by an atomic absorption spectrophotometer (GBC Avanta, Australia). For quality control, a known concentration Pb-standard solution (20 µg/dL) was added to the sample and analyzed. The recovery rate was found to be 100% with less than 5% relative standard deviation for three replicates.

###  Bone formation markers

Serum levels of ALP, BAP, and OC were used as bone growth markers. The International Federation of Clinical Chemistry (IFCC) method was used to estimate the ALP. The BAP activity was determined using the phenylalanine inhibition technique^33^. Serum OC concentration was measured using the enzyme-linked immune-absorbent assay method (YH Bio search Laboratory, China). The absorbance of standards and samples were measured using Thermo Scientific Multiskan EX-reader (USA) at 450 nm. The detection range and sensitivity of the method were obtained at 0.5-150 ng/mL and 0.026 ng/mL, respectively. Serum OC quantity was expressed as ng/mL.

###  Bone resorption markers

 The Pyr, DPyr, and TACRP-5 b in serum were estimated by enzyme-linked immunosorbent assay (ELISA), and HyP in urine was measured by spectrophotometric method. Serum Pyr and DPyr were measured using the kit supplied by YH Bio search Laboratory, China. The absorbance of standards and samples were measured using Multiskan EX-reader (Thermo Scientific, USA) at 450 nm. The standard calibration curve was prepared in the range of 0.5-200 ng/mL for Pyr and used to determine unknown sample concentration with a sensitivity of 0.024 ng/mL. The assay range for DPyr method is 5-1000 nmol/L with a sensitivity of 5 nmol/L, and the results were expressed as nmol/L.

###  Serum Tartrate-​Resistant Acid Phosphatase -5b

The estimation of serum TRACP-5b was conducted using Sarvari et al. ^34^ method in which the serum samples were diluted tenfold in distilled water and incubated at 37°C for 1 h. An aliquot of 50μl diluted sample was added to 50μl of substrate solution in a Microplate, and the reaction was carried out at 37°C for 1 h. The reaction was stopped by the addition of 50 μl of 1 M NaOH. A standard calibration curve was prepared in the range of 5-25 μg/ml using the p-nitrophenol solution in 0.05 M NaOH to determine unknown concentrations. The absorbance of standards and samples was assessed using a Multiskan microplate reader (Thermo Scientific, USA)) at a wave length of 405 nm, and the results were expressed as U/L. One unit (U) of TRACP-5b activity is defined as the amount of enzyme required to hydrolyze one micromole of p-nitrophenyl phosphate (pNPP) per minute at 37°C.

###  Urinary-HYP

The UHyP was estimated using the modified Neuman and Logan method^35^. The hydroxyproline in urine reacted with CuSO_4_ and H_2_O_2_ in an alkaline solution and produced pyrroline-4-carboxylic acid which is on acidification converted into pyrrole-2-carboxylic acid. The product was condensed with p-dimethyl amino benzaldehyde to obtain a red-colored complex, and the absorbance was measured at 540 nm using a spectrophotometer (Elico-SL159, India). The urinary hydroxyproline was expressed as µg/gram of creatinine.

###  Statistical analysis

The data analysis was performed in SPSS software (version 20). The normality of continuous variables was evaluated using the Shapiro-Wilk test. Study and control subjects were compared in terms of such variables as age, Body mass index (BMI), systolic blood pressure (SBP), diastolic blood pressure (DBP), and BLLs using the student t-test. The non-normal distributed continuous variables, such as BTMs, between the subjects with and without MSDs were compared using the Mann-Whitney U test. The Chi-square test was also employed to compare the subjects in the study and control groups in terms of the frequency distribution of smoking, alcohol consumption, as well as pain and discomfort in the musculoskeletal system. The general linear model (GLM) with multivariate analysis was used to assess the effect of age, BMI, experience, BLLs, and BTMs on pain and discomfort of the musculoskeletal system. A *P*-value of less than 0.05 was considered statistically significant.

## Results

The demographic characteristics of study and control subjects are presented in Table 1. The mean scores of age, SBP, DBP, BMI, as well as the frequency distribution of alcohol consumption and smoking habits among study subjects were found to be similar to those obtained in controls. The BLLs were significantly higher among the study group (*P*<0.001), as compared to controls. World Health Organization (WHO) has identified a BLLs threshold of 40 µg/dL for occupationally exposed workers (1980). Moreover, 49 (27.84%) cases in the study group were higher than the WHO threshold value for BLL; nonetheless, BLLs were below the WHO limit in the control group ^36^.

Pain and discomfort in different parts of the body in study and control subjects are illustrated in Table 2. The study and control subjects were compared in terms of pain and discomfort in the musculoskeletal system using the Chi-square test. The proportion of pain and discomfort in the neck (*P*=0.017), shoulders (*P*=0.014), elbows (*P*=0.009), wrist/hands (*P*=0.015), upper back (*P*=0.017), low back (*P*=0.001), knee (*P*=0.049) and ankle/foot (*P*=0.002) were significantly higher in study subjects, as compared to controls. The most commonly reported pain and discomfort in the musculoskeletal system were observed at the lower back, followed by knee, shoulders, neck, ankle/feet, wrist/hand, elbows, upper back, and hips/thighs.

**Table 1:** Demographic detail of study and control subjects

**Continuous variables**	**Study (176)**		**Controls (80)**		***P*** **-value**
	**Mean**	**SD**	**Mean**	**SD**	
Age (yr)	36.6	4.0	37.4	10.0	0.361
Body mass index (Kg/m^2^)	25.7	2.9	25.3	3.4	0.339
Occupational Pb exposure (yr)	13.3	3.3	0.0	0.0	-
Diastolic Blood Pressure (mmHg)	77.7	10.7	74.8	12.7	0.059
Systolic Blood Pressure (mmHg)	127.8	14.6	127.5	19.1	0.890
Blood lead levels (µg/dL)	32.3	12.0	20.1	6.0	0.001
**Categorical variables**	**Number**	**Percent**	**Number**	**Percent**	***P*** **-value**
Alcohol consumption	95	54	39	49	0.437
Smoking	38	22	19	24	0.700

**Table 2:** Pain and discomfort of the musculoskeletal system of study and control subjects

**Pain And Discomfort In The Body Region**	**Control (N=80), n (%)**	**Study** **(N=176), n (%)**	***P*** **-value**
Neck	3 (3.75)	24 (13.63)	0.017
Shoulders	4 (5.00)	28 (15.91)	0.014
Elbows	0 (0.00)	14 (7.95)	0.009
Wrist/Hands	1 (1.25)	17 (9.65)	0.015
Upper Back	0 (0.00)	12 (6.81)	0.017
Lower Back	7 (8.75)	58 (32.95)	0.001
Hips/Thighs	1 (1.25)	8 (4.54)	0.185
Knees	12 (15.00)	46 (26.13)	0.049
Ankle/Feet	0 (0.00)	20 (11.36)	0.002

The median BLLs and BTMs in subjects with or without pain and discomfort in the musculoskeletal system are displayed in Table 3. Higher BLLs were noted in subjects with pain and discomfort in the musculoskeletal system, as compared to those without such pain. Significantly higher BLLs were reported in subjects with pain and discomfort in the upper back (*P*<0.001), lower back (*P*<0.001), and ankle/foot (*P*=0.011), as compared to those without respective pain and discomfort in the musculoskeletal system. In addition, the subjects with pain in elbows (*P*=0.033) and ankle/foot (*P*=0.042) had significantly lower OC, in comparison with those without pain and discomfort. Furthermore, the subjects with pain and discomfort in the neck (*P*=0.049) and shoulders (*P*=0.023) had significantly lower levels of collagen degradation products, such as serum DPyr, as compared to those without these ailments. Subjects with pain and discomfort in the shoulders (*P*=0.035) and lower back (*P*=0.036) had significantly higher levels of UHyp, as compared to those without respective compliant.

**Table 3:** Median levels of blood lead and bone turnover markers among subjects with or without musculoskeletal pain

**Pain & discomfort** **Body region**	**BLLs** **(µg/dL)**	**Bone formation**			**Bone resorption**			
		**ALP** **(U/L)**	**BAP** **(U/L)**	**OC** **(ng/mL)**	**Pyr** **(ng/mL)**	**Dpyr** **(nM /L)**	**TRACP5b** **(U/L)**	**UHyP** **(µg/ gcre )**
Neck								
No (229)	25.00	86.00	37.00	13.00	30.00	236.00	2.50	5.00
Yes (27)	29.00	97.00	38.00	12.00	29.00	205.00	3.00	5.60
Shoulders								
No (224)	25.00	86.00	37.00	14.00	30.00	238.00	2.60	5.00
Yes (32)	26.50	92.00	34.50	12.00	29.00	205.00	2.65	6.25
Elbows								
No (242)	25.00	87.50	37.00	13.00	29.50	235.00	2.65	5.00
Yes (14)	30.00	74.00	33.50	11.00	31.00	216.50	2.30	5.05
Wrist/hands								
No (238)	25.50	86.00	37.00	13.00	29.50	235.50	2.60	5.00
Yes (18)	30.50	92.50	40.00	11.00	31.00	212.50	2.85	5.05
Upper back								
No (244)	25.00	86.00	37.00	13.00	30.00	235.50	2.60	5.00
Yes (12)	36.50	103.00	36.50	12.00	30.50	213.50	2.50	6.40
Lower back								
No (191)	24.00	86.00	37.00	14.00	30.00	235.00	2.60	5.00
Yes (65)	32.00	87.00	39.00	12.00	30.00	235.00	3.00	5.20
Hips/Thighs								
No (247)	25.00	86.00	37.00	13.00	30.00	235.00	2.60	5.00
Yes (09)	28.00	109.00	36.00	11.00	35.00	235.00	3.00	5.30
Knees								
No (198)	25.00	86.00	35.00	12.50	29.00	237.00	2.60	5.00
Yes (58)	29.00	88.00	38.50	12.00	31.00	231.50	3.00	5.00
Ankles/foot								
No (236)	25.00	86.00	37.00	13.00	30.00	235.00	2.65	5.00
Yes (20)	35.00	88.50	37.50	12.00	29.50	242.50	2.50	5.00

BLLs=blood lead levels, ALP=Alakline phosphatse, BAP=Bone alkaline phosphatase,OC=Osteocalcin, Pry=Pyridinoline, DPyr=deoxypyridinoline, TRACP-5b=tartrate-resistant acid phosphatase-5b,UHyP=urinary hydroxyproline

The median BLLs and BTMs among subjects with pain and discomfort in musculoskeletal systems of the upper body, as well as lower and upper extremity are reported in Table 4. The subjects with pain in the lower extremity had significantly higher BLLs (*P*=0-001), as compared to cases without the respective disorder. Although lower levels of serum OC were observed among subjects with pain and discomfort in the musculoskeletal system, it was not statistically significant. The collagen degradation products, such as serum DPyr, were significantly lower (*P*=0.005), and UHyp was significantly higher (*P*=0.006) among subjects with pain and discomfort in the upper body part of the musculoskeletal system.

**Table 4:** Median levels of blood lead and bone turnover markers among subjects with or without pain and discomfort of the upper body part, lower and upper extremities

**Area of body (number)**	**BLLs** **µg/dL**	**Bone formation**			**Bone resorption**			
		**ALP** **U/L**	**BAP** **U/L**	**OC** **ng/mL**	**Pyr** **ng/mL**	**DPyr** **nM /L**	**TRACP5b** **U/L**	**UHyP** **µg/ gcre**
Upper body (Neck, shoulder, upper back)								
No (200)	25.00	86.00	37.00	13.00	30.00	238.00	2.55	5.00
Yes (56)	29.00	89.00	35.00	12.50	29.00	205.00	2.70	5.65
Upper extremity (Elbow, wrist, hand)								
No (231)	25.00	86.00	37.00	13.00	29.00	235.00	2.60	5.00
Yes (25)	32.00	90.00	38.00	11.00	31.00	216.00	2.70	5.00
Lower extremity (Thighs, knee and Ankle/foot)								
No (173)	24.00	86.00	35.00	14.00	29.00	236.00	2.60	5.00
Yes (83)	31.00*	88.00	39.00	12.00	31.00	234.00	3.00	5.00

BLLs=blood lead levels, ALP=Alakline phosphatse, BAP=Bone alkaline phosphatase,OC=Osteocalcin, Pry=Pyridinoline, DPyr=deoxypyridinoline, TRACP-5b=tartrate-resistant acid phosphatase-5b,UHyP=urinary hydroxyproline

The details of multivariate regression analysis are presented in Table 5. The general linear model (GLM) with multivariate analysis was used to assess the association between dependent and independent variables. In this model, the bone remodeling (bone formation & bone resorption) markers were employed as continuous dependent variables, while pain and discomfort in the skeletal system (neck, shoulders, elbows, wrist/hands, upper back, lower back, hips/thighs, knees and ankles/foot) were used as fixed factors or categorical independent variables after controlling for covariates of age, BMI, experience, smoking, alcohol consumption and blood pressure (SBP and DBP). The association between dependent and independent variables was evaluated using Wilks' Lambda (A), exact statistics (F), probability (P), and effect size (partial eta squared). The pain and discomfort in the upper back (Wilks' A=0.983, F=2.558, *P*=0.015 & partial ᵑ2=0.09), blood lead levels (Wilks'A=0.175, F=1.163, P=0.039&partial ᵑ2=0.22), and experience (Wilks' A=0.859, F=4.399, *P*=0.001 and partial ᵑ2=0.14), was significantly associated with combined bone remodeling markers after controlling for covariates.

**Table 5:** Details of multivariate regression analysis

**Effect**	**V alues of ** **Wilks' lambda**	**F**	***P*** **-value**	**Partial eta squared**
Neck	0.974	0.727	0.649	0.026
Shoulders	0.980	0.541	0.803	0.020
Elbows	0.966	0.947	0.472	0.034
Wrist/ Hands	0.983	0.462	0.861	0.017
Upper back	0.913	2.558	0.015	0.087
Lower back	0.978	0.604	0.752	0.022
Hips/ Thighs	0.960	1.107	0.360	0.040
Knees	0.981	0.517	0.821	0.019
Ankle/Feet	0.936	1.838	0.082	0.064
blood lead levels	0.175	1.163	0.039	0.220
Age	0.953	1.305	0.250	0.047
Body mass index (kg/m^2^)	0.942	1.632	0.129	0.058
Experience	0.859	4.399	0.001	0.141
Smoking	0.966	0.943	0.475	0.034
Alcohol consumption	0.957	1.189	0.311	0.043
Systolic blood pressure (mmHg)	0.960	1.109	0.359	0.040
Diastolic blood pressure (mmHg)	0.941	1.671	0.118	0.059

## Discussion

According to WHO, Pb exposure accounted for 1.06 million deaths and 24.4 million years of healthy life lost (DALY) ^37^. Furthermore, chronic Pb-exposure and chronic Pb-poisoning are associated with deleterious systemic effects, including the nervous system, cardiovascular, hemopoietic, renal, reproductive, skeletal, and gastrointestinal ^38,39^. The mechanism of the effect of Pb-toxicity on dysfunction in different organs is reported due to displacement of divalent metal ions ^40^, oxidative damage ^41^, inhibition of thiol groups in proteins ^42^, inhibition of enzymes in heme biosynthesis pathway ^43^, and epigenetic modifications ^44^.

The present study evaluated the association of BTMs (formation & resorption) with pain and discomfort in the musculoskeletal system among long-term Pb-exposed workers in a Pb-battery plant. The blood Pb was estimated to assess Pb-exposure ^45^. We witnessed significantly higher BLLs with long-term Pb exposure. Moreover, the obtained results pointed to a significantly high prevalence of pain and discomfort in the lower back, followed by knees, shoulders, and neck among Pb-exposed workers. Construction workers ^46^, workers of garment factories ^47^, gold miners ^48^, and physical therapists ^49^ also reported lower back pain and discomfort. They also complained about pain and discomfort in the neck, knees, shoulders, and upper back. The possible reasons for these work-related MSDs are extreme trunk flexion, repetitive motion, kneeling, heavy physical activity, and pushing and pulling of loads.

Lower bone formation (serum OC) was also detected with pain and discomfort in elbows and ankle/foot. The pain and discomfort in the musculoskeletal system of the neck, shoulders, and lower back were associated with bone resorption markers. In the present study, it was noted that BLLs in the study group were significantly higher, as compared to those in the control group. A recent study also reported significantly higher BLLs in similar occupational group ^50^ even at low levels of Pb-exposure, the musculoskeletal system is susceptible to Pb-toxicity ^21^. Based on the literature review, Pb-intoxicated animals had noxiousness in the peripheral motor system, muscular inactivity, and myopathy changes ^51^. Occupational Pb exposure is associated with lower BMD ^52^, osteoporosis ^53^, and high bone resorption markers ^5,54^. Bilateral weakness of proximal limb muscles, including shoulders, elbow, and hips, were reported with severe Pb poisoning ^55^. Nelson et al. ^56^ pointed out that Pb-exposure was primarily associated with bone and calcium turnover, as well as cartilage metabolism. In the current study, it was observed that BLLs were significantly associated with pain and discomfort in the musculoskeletal system of the upper back, lower back, and ankle/foot. Long-term Pb-intoxication can cause motor neuron disease (MND), which is characterized by a decrease in the amplitude of the motor response, spinal motor neuron degeneration, axonal motor loss, and atrophy of muscle ^57^. The present study strengthened the previous observations regarding the association of chronic Pb-exposure with susceptibility of lower limbs, lower back, and upper back pain and discomfort in the musculoskeletal system.

The evaluation of BTMs is usually performed for the detection of early osteoporosis ^12^. Based on related studies, the pain and discomfort in the musculoskeletal system are multimorbidities ^58^, and these ailments are characterized by inflammation, pain, and motor dysfunction ^59^. It was suggested that the markers of inflammation, cell stress, collagen synthesis, and degradation can serve as predictors for work-related musculoskeletal injury ^60^. Ravibabu et al. ^29^ pointed to the association of Pb-exposure with inflammatory markers and MSDs of lower limbs. The current study examined the relationship of Pb-exposure with pain and discomfort in the musculoskeletal system and BTMs. As documented in the literature, cartilage break down ^61^ and lower cartilage thickness ^62^ are associated with knee osteoarthritis. The present study assessed the relationship between bone resorption markers and discomfort in the musculoskeletal system among workers. The examined parameters, such as serum Pry, DPry, and UHyP, were used as collagen fragment markers and serum TRACP-5b, as a marker of osteoclast activity. In the current study, lower levels of serum Pyr, DPyr, and higher levels of serum TRACP-5b and UHyP were observed in workers who had pain and discomfort in the neck and shoulders. These observations were similar to the findings of a study conducted by Cucu et al. ^63^. Furthermore, it was documented that neck/shoulder pain is associated with raised inflammation markers ^64^. In this study, we observed that the pain and discomfort of the lower back were significantly associated with a higher excretion of urinary HyP, which is an indicator of collagen breakdown. Previous studies confirmed that pain and discomfort in the musculoskeletal system are characterized by reduced vascularity, loss of grip strength, muscle pain, muscular atrophy, and enhanced collagen degradation with lower muscle mass ^65,66^. The findings of the present study also confirmed that the collagen fragments are susceptible to pain and discomfort in the neck and shoulders.

Carp et al. ^60^ also liked the elevated levels of collagen turnover with pain and discomfort in the musculoskeletal system. In the present study, it was noted that the pain and discomfort in the ankle/foot and elbows were associated with lower serum OC, which is a marker of bone formation and osteoblast activity. Taylan et al. ^67^ also pointed out a significantly lower concentration of serum osteoprotegerin with ankylosing spondylitis, which is characterized by a decrease in osteoblastic activity. The results of this study indicated that the osteoblast activity in the form of bone formation was significantly lower in workers who had pain and discomforts in the knee and elbows. In the same context, Bhilet et al. ^68^ reported increased levels of urinary-CTX-II in osteoarthritis of knee joints. No significant association was detected between knee pain and collagen BTMs among workers in the current study. Along the same lines, Gielen et al. ^69^ stated that the BTMs could not predict accelerated hip bone loss. In the current study, it was indicated that hip/thighs pain and discomfort were not significantly associated with BTMs. In line with the results of the present study, Masonet al. ^28^ reported that lower limb pain and discomfort were significantly associated with cartilage markers and urinary C-telopeptide of collagen II.

 The present study also assessed the association of BTMs with three groups of pain and discomfort in the musculoskeletal system among workers. The three groups of pain and discomfort of the body areas are categorized into upper body, upper extremity, and lower extremity. The pain and discomfort in the upper body include neck, shoulder, and upper back. The pain and discomfort in elbows and wrist/hands were regarded as an upper extremity. The pain and discomfort in thighs/hips, knees, and ankle/foot were considered the lower extremity. The BLLs were higher in workers who had pain and discomfort in the upper and lower extremities. A significant increase was observed between lower extremity and BLLs. The bone resorption markers, such as serum DPyr and UHyP, were significantly associated with pain and discomfort in the upper body.

## Conclusion

 As evidenced by the obtained results, Pb-exposure was associated with pain and discomfort in musculoskeletal systems of the upper back, lower back, and ankle/foot. Lower bone formation (serum OC) was associated with pain and discomfort in the elbows and ankle/foot musculoskeletal system. Bone resorption markers were associated with pain and discomfort in the musculoskeletal system of the neck, shoulders, and lower back. The results of the present study confirmed that long-term Pb-exposure and BTMs (Formation and resorption) were associated with pain and discomfort in the musculoskeletal system. These markers are of great help in better estimation of negative effects of chronic Pb-exposure to undertake appropriate preventive measures at the earliest.

## Acknowledgments

 The authors are grateful to the Director, ICMR-National Institute of Occupational Health, for his support and encouragement in conducting this study. Authors are thankful to Dr. Raju N, AdepuVinay Kumar, N. Thara, and Miss. Shabrin Fatima for their technical assistance. Last but not least, the authors are grateful to the study participants, who willingly co-operative with the study.

## Conflict of interests

 The authors declare that they have no conflict of interest.

## Funding

 Indian Council of Medical Research.

## Highlights

- The present study aimed to assess the association between musculoskeletal pain and bone turnover markers in lead (Pb)-exposed workers.
- Pb exposure was significantly associated with lower back, upper back, and ankle/foot pain.
- Bone formation marker: Lower serum osteocalcin was significantly associated with elbows and ankle/foot pain.
- Bone resorption marker: Lower serum deoxypyridinoline was significantly associated with neck and shoulder pain.
- Bone resorption marker: Higher hydroxyproline in urine was significantly associated with shoulder and lower back pain.
- Multivariate regression analysis indicated that the upper back, blood lead, and years of Pb exposure were significantly associated with bone turnover markers.
